# Close encounter of the covalent kind: Inhibiting MCL1’s proapoptotic activity with covalent inhibitors

**DOI:** 10.1038/cddiscovery.2016.94

**Published:** 2017-01-23

**Authors:** Guillaune Lessene

**Affiliations:** 1 Chemical Biology Division, The Walter and Eliza Hall Institute, Parkville, VIC, Australia; 2 Department of Pharmacology and Therapeutics, The University of Melbourne, Melbourne, VIC, Australia

Targeting the BCL-2 family proteins to combat cancer is now a *fait accompli*. With the recent FDA approval of venetoclax for the treatment of 17p-deleted Chronic Lymphoma Leukemia (CLL), patients suffering from a subtype of the disease associated with very poor prognosis can now access an entirely new class of drugs, which works by re-establishing apoptotic cell death in cancer cells.^1^ This achievement validates the bold idea that arose from the discovery of BCL-2’s role in cancer in the early 1990’s.^2^ Since then, much has been discovered about the BCL-2 family and its members,^3^ which are divided in two main subgroups: one is characterized by its prosurvival activity (that is, preventing cell death) and includes proteins such BCL-2 itself, BCL-X_L_ or MCL1. A second subgroup promotes cell death and is itself divided into the executioners BAX/BAK (and BOK), which form pores on the mitochondrial outer membrane to allow apoptogenic factors such as cytochrome *c* to be released leading to caspase activation and cell demolition. The other group of proapoptotic proteins, the so-called ‘BH3 only proteins’ (for example, BIM, BAD, NOXA), operate upstream of the prosurvival proteins and act as sensors of cellular stress, bind to prosurvival BCL-2 family proteins, resulting in the release of proapoptotic activity of BAX and BAK. This cascade of inhibition between pro- and anti-apoptotic proteins is mediated by protein-protein interactions through helix-in-groove interactions.^3^ Overexpression of prosurvival proteins, which results in a block of the apoptotic response, is an important trait of most cancers. Reestablishing this response with small molecule drugs, coined ‘BH3-mimetics’ (that is, replicating the functional activity of BH3-only proteins) has been a highly challenging drug discovery goal.^4^

Designing highly potent and selective agents that disrupt the large and mainly hydrophobic interfaces between the BCL2 proteins is not an easy task and has forced medicinal chemists to venture into unchartered territories of drug-like parameters. ABT-199/venetoclax, despite its size and properties, is an orally available drug with a remarkable efficacy profile.^1^ Because apoptosis lies at the center of a multitude of biological pathways, it is has also been surprisingly difficult to demonstrate that these compounds induce cell death by directly interacting with the prosurvival proteins.^4^ A significant number of putative BCL-2 inhibitors have thus been reported, yet it is now clear that most of them act through an indirect mechanism.^5^ To date, only a handful of small molecules comply with criteria defined for genuine BH3-mimetics,^4,5^ that is, induce apoptosis by directly interacting with one or several pro-survival proteins.

In this evolving landscape, MCL1 has remained a recalcitrant target fraught with its own challenges (for example, a binding groove less amenable to screening and design). As a result, the discovery of selective inhibitors of MCL1 has lagged considerably compared with its congeners BCL-X_L_ and BCL-2.^4^ From a therapeutic point of view, the pharmacological inhibition of MCL1 clearly is an attractive prospect: reliance of MCL1 may be a characteristic of up to 25% of all cancers.^6^ Cancers such as AML,^7^ multiple myelomas and *myc*-driven lymphomas^8^ are amongst the malignancies shown to rely on MCL1 for survival. However, another challenge arising from MCL1’s role in biology probably diminished the enthusiasm of even the most daring drug discoverers. Genetic knockout studies painted a bleak scenario for MCL1 inhibition: very early embryonic lethality, toxicities to organs such as the heart, liver or various hematological compartments.^3^ This specter of toxicity was partly relieved through some recent studies using heterozygote animals that suggested that full therapeutic potential could be achieve with the removal of only one *mcl1* allele with little impact on the animal health,^7–9^ suggesting that a potential therapeutic window could be achieved.

Because of the challenges associated with targeting the BCL-2 family of proteins in general and MCL1 in particular, scientists have been forced to devise new strategies to design small molecule BH3-mimetics. In their paper, Akcay *et al.*,^9^ a group from Astra Zeneca, describe an interesting approach based on the formation of a covalent but reversible bond between their inhibitor and MCL1 (Figure 1). While the idea of covalent inhibitors (reversible or irreversible) is not novel (largely explored in the field of kinase), it has so far never been used to target the BCL2 family of proteins with small molecules (a close example has been recently described with a reactive stapled peptide^10^). Akcay *et al.* use a recently described formyl boronic acid moiety, which acts as the reactive group to specifically and preferentially create a covalent bond with lysine residues.

As a proof of concept for their approach, they took advantage of a known class of MCL1 inhibitors and available X-ray structures.^11,12^ With this information in hand, they elegantly designed new inhibitors placing the reactive moiety in close proximity of Lysine 234 located in the BH3 binding groove.

Beyond the innovative approach, the strength of the paper resides also in the efforts made to characterize the activity of these reactive compounds. The authors first demonstrate that the inhibitor bearing the best reactive moiety have enhanced binding affinity (4.2 nM compared with 383 nM for the parent compound) associated with significant induction of apoptosis (measured through caspase activation) in a cell line relying on MCL1 for survival (MOLP-8). This activity was then confirmed using a panel of myelomas with various MCL1 dependencies.

BAX/BAK dependency is one of the hallmarks of intrinsic, mitochondrial apoptosis.^3^ Interestingly, Akcay *et al.* demonstrate that the activity of their reactive compound seems mainly mediated by BAK as SiRNA-mediated knockdown of BAK led to a significant decrease in activity. Through a series of binding experiments using Surface Plasmon Resonance, they provide information regarding the reaction kinetics with the lysine residue. Finally, using MS experiments together with the expression of a variant of MCL1 lacking the key lysine residue Lys324, they prove that the reactive compound forms an adduct with this amino acid located in the groove, as intended. Altogether, this paper demonstrates that reactive inhibitors of BCL-2 family proteins can be developed to improve activity of the parent compound: the single agent activity of the best analogue presented by Akcay *et al.* is far better than that of an unreactive analogue.^13^ This is a notable result because designing a successful reactive moiety is not trivial even when structural information is available. Will this type of reactive compound be suitable for clinical development? Only time will tell, especially since no *in vivo* data are presented in this study. Notably, a recent publication on potent MCL1 inhibitor shows that such a compound need not be reactive to achieve very high binding affinity and potent single agent activity.^11^ At the molecular level, the paper also raises questions about the impact of these compounds on MLC1 stability. Indeed, MCL1 levels are tightly regulated *via* multiple mechanisms, in particular proteasomal degradation, making MCL1 a short-lived protein.^3^ Will a reactive inhibitor have the same stabilizing effect as that observed with S63845? How does the formation of a covalent bond with MCL1 play out *in vivo* considering MCL1’s limited half-life?

Despite these fascinating questions, the paper from Akcay *et al.* is a clear advance in the field of BH3-mimetics as it provides a new type of weapon in a slowly expanding armamentarium. We can expect more examples and hopefully drugs derived from this concept.

## Figures and Tables

**Figure 1:**
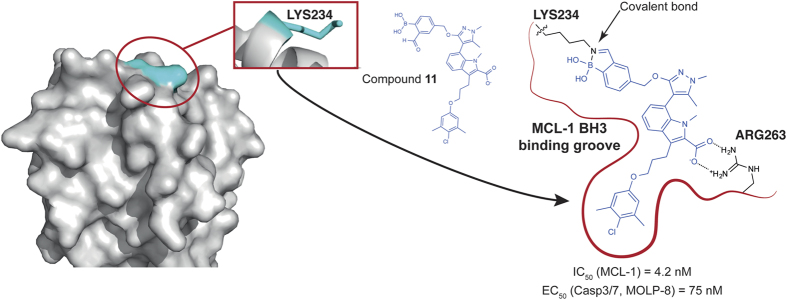
Upon binding into the BH3-binding groove, compound **11** reacts with LYS234 to form a covalent bond. The design of compound **11** was based on previously reported MCL1 inhibitors, which engage MCL1 by forming a deep pocket into MCL1 and creating a charged interaction with ARG263. The covalent bond in compound **11** increases the stability of the complex between the inhibitor and the protein (IC_50_=4.2 nM) and, as a consequence, induces the activation of caspases 3/7 activation in MCL1 dependent MOLP-8 cells (IC_50_=75 nM). On the left, MCL1 is shown as surface representation (PDB entry: 3WIX).

## Notes

GL is an employee of the Walter and Eliza Hall Institute of Medical Research, which receives research funding and milestone payments in relation to venetoclax (ABT-199). GL also receives research funding from Servier.

## References

[bib1] Roberts AW et al. New Eng J Med 2016; 374: 311–322. 26639348 10.1056/NEJMoa1513257 PMC7107002

[bib2] Vaux DL, Cory S, Adams JM. Nature 1988; 335: 440–442. 3262202 10.1038/335440a0

[bib3] Czabotar PE et al. Nat Rev Mol Cell Biol 2014; 15: 49–63. 24355989 10.1038/nrm3722

[bib4] Lessene G, Czabotar PE, Colman PM. Nat Rev Drug Discov 2008; 7: 989–1000. 19043450 10.1038/nrd2658

[bib5] Soderquist RS, Eastman A. Mol Cancer Ther 2016; 15: 2011–2017. 27535975 10.1158/1535-7163.MCT-16-0031 PMC5010924

[bib6] Beroukhim R et al. Nature 2010; 463: 899–905. 20164920 10.1038/nature08822 PMC2826709

[bib7] Glaser SP et al. Genes Dev 2012; 26: 120–125. 22279045 10.1101/gad.182980.111 PMC3273836

[bib8] Kelly GL et al. Genes Dev 2014; 28: 58–70. 24395247 10.1101/gad.232009.113 PMC3894413

[bib9] Akcay G et al. Nat Chem Biol 2016; 12: 931–936. 27595327 10.1038/nchembio.2174

[bib10] Huhn AJ et al. Cell Chem Biol 2016; 23: 1123–1134. 27617850 10.1016/j.chembiol.2016.07.022 PMC5055752

[bib11] Kotschy A, S et al. Nature 2016; 538: 477–482. 27760111 10.1038/nature19830

[bib12] Bruncko M et al. J Med Chem 2015; 58: 2180–2194. 25679114 10.1021/jm501258m

[bib13] Leverson JD et al. Cell Death Dis 2015; 6: e1590. 25590800 10.1038/cddis.2014.561 PMC4669759

